# Psychometric properties of the Danish Parental Stress Scale: Rasch analysis in a sample of mothers with infants

**DOI:** 10.1371/journal.pone.0205662

**Published:** 2018-11-07

**Authors:** Maiken Pontoppidan, Tine Nielsen, Ingeborg Hedegaard Kristensen

**Affiliations:** 1 Department of Health, VIVE—The Danish Center for Social Science Research, Copenhagen, Denmark; 2 Department of Psychology, University of Copenhagen, Copenhagen, Denmark; 3 Department of Public Health, Section for Nursing, Aarhus University, Aarhus, Denmark; Leibniz Institute for Educational Trajectories, GERMANY

## Abstract

The Parental Stress Scale (PSS) was developed as a short measure of perceived stress resulting from being a parent. The current study examined the psychometric properties of the Danish version in a sample of 1110 mothers of children aged 0 to 12 months using Rasch models. Emphasis was placed on the issues of uni-dimensionality and absence of differential item functioning relative to the age and educational level of the mothers. Results showed that no adequate fit could be established for the full PSS scale with 18 dichotomized items. Further analyses showed that items 2 and 11 had to be eliminated from the scale, and that the remaining items did not make up a unidimensional PSS scale, but two subscales measuring different aspect of parental stress: a 9-item scale measuring parental stress and a 7-item scale measuring lack of parental satisfaction. Fit to the Rasch model could not be established for any of the two subscales. For the parental stress subscale, we found evidence of local dependence for four item pairs (3 and 4, 9 and 10, 10 and 16, 12 and 16), as well as evidence of two items functioning differentially: item 16 relative to level of education, and item 3 relative to both age and educational level. For the lack of parental satisfaction subscale, we found evidence of local dependence between some two pairs (1 and 17, 17 and 18), but no evidence of differential item functioning. Both subscales fit graphical loglinear Rasch models adjusting for local dependence and differential item functioning. Plotting the adjusted subscale scores against one another showed that the two-scale solution provides additional information, as some mothers are stressed but not lacking in parental satisfaction.

## Introduction

Having a child is both a rewarding and taxing experience and parental stress is a normal part of being a parent [1–4]. Parental stress can be defined as “a set of processes that lead to aversive psychological and physiological reactions arising from attempts to adapt to the demands of parenthood” [5]. Levels of parental stress are not static but fluctuate according to the developmental stages of the child and the demands the parents face [6].

To develop a healthy psychosocial development and a secure attachment bond with caregivers, infants need warm and sensitive caregivers [7,8]. Caring for an infant can be challenging, particularly for parents of infants who show persistent crying or sleep disturbances [9–11]. Whereas short periods of stress can be beneficial in many ways, long-term and/or chronic stress can have deleterious effects on the infant [4], and cause both imbalance of the neural circuitry in the brain [12] and detrimental effects on the immune system [13].

Parents experiencing high stress levels may struggle to meet some of the basic psychosocial needs of their infant and engage in less growth-promoting parenting behaviors which may then impact child development [6,14]. Parents of infants without any obvious difficulties may also experience parental stress, due to differences in coping with the many changes in life circumstances for first-time parents, and the continued effect of being a parent on other aspects of life such as relationship, sexuality, and work [15]. Parental stress is related to poverty, loneliness, depression, anxiety, and substance abuse problems and has been linked to child behavior problems and a less responsive and more harsh and punitive parenting style [4,15–22]. In order to identify parents that experience high levels of parental stress and possibly intervene, it is important to be able to measure parental stress in a valid and reliable way.

The Parental Stress Scale (PSS) measures “individual differences in the level of stress associated with raising children” [1]. It is centered on the individual’s perception of stress rather than the actual sources of stress. The PSS addresses the dichotomy of parenting; that it is both stressful and satisfying by including both negative and positive aspects in the items, and then reversing the positive ones to make the overall scale a measure of stress.

Berry and Jones found that the English PSS showed adequate reliability (Cronbach’s alpha = .83) as well as adequate reliability over time (test-retest correlation = .81 over six weeks). Other studies found corresponding reliability results for different language versions: Cronbach’s alpha was reported to be .89-.90 for the Chinese version with 16 and 17 item, respectively [23,24], .76 and .77 for two subscales in the Spanish 13 item version [25], .60 and .79 for two subscales in the Portuguese 16 item version [26], and .73 for the Urdu version with 19 items [27]. Criterion validity for the PSS has been supported by studies finding positive correlations with the following measures: (1) Perceived Stress Scale [1,28], (2) the total score on the Parenting Stress Index [1,29], (3) anxiety scores (State-Trait Anxiety Inventory [25,30]), (4) depression symptoms (Beck Depression Inventory (BDI) [25,31]), and (5) parental attitude (Index of Parent-Child Relations [23,32]). However, as different language versions of the PSS with both a different number of items and different measurement properties were used in these studies, they should be compared cautiously.

Previous research on the PSS has aimed at distinguishing between groups that are expected to show average parental stress levels and groups expected to show high parental stress levels. Thus studies have compared parental stress scores for (1) mothers with children in treatment for emotional/behavioural problems and mothers with children not in treatment [1], (2) mothers with children with developmental disabilities who were receiving special education and mothers with children without developmental disabilities [1], (3) parents participating in parent education programs (adjusted group) and parents who sought counselling (maladjusted group) [23], and (4) parents with children in primary schools and parents with children with attention deficit hyperactivity disorder (ADHD) [24]. Several studies support Berry and Jones’ original proposal that the parental experience is a dichotomy of stress and satisfaction. Therefore, we propose that more research is needed in order to ascertain whether the PSS should be used as a single unidimensional measure of parental stress, such as is the practice when summing up one total score across the stress items and the reversed satisfaction items, before the criterion validity can be more accurately assessed.

We have been able to identify five previous validity studies of the PSS, including the original development study by Berry and Jones [1]. Berry and Jones, using only exploratory factor analysis identified four and not two dimensions of the PSS. However, these are simply subdivisions of the parental stressors and parental satisfaction. The remaining studies employ different language versions of the PSS, and use different statistical methods: Oronoz, Alonso-Arbiol and Balluerka [25] employed a Spanish version of the PSS and again only exploratory factor analysis. They identified the two main dimensions of the PSS. Brito and Faro [26] employed a Portuguese PSS and also used exploratory factor analysis to identify two dimensions covering the two main aspects of parental stress.

Cheung [23] and Leung and Tsang [24] both employed the same Chinese version of the PSS. Cheung used an exploratory principal component analysis and identified the two main dimensions of the PSS. Leung and Tsang [24] used the rating scale version of the Rasch model [33] with the Chinese version of the PSS. They found that the PSS fit a single unidimensional scale measuring parental stress. Thus the majority of studies, with the exception of Leung and Tsang [24], have suggested that the PSS is made up of two separate dimensions though not with the exact same items in them; parental stressors or stress as one subscale and parental satisfaction or lack of satisfaction as the other subscale. Furthermore, all studies agreed that item 2 *(There is little or nothing I wouldn't do for my child(ren) if it was necessary)* should be excluded. Each of the studies do, however also suggest elimination of other items. Thus, the two-dimensionality of the PSS is still not formally tested, and the psychometric properties of the two subscales remain to be studied using the thorough approach of Rasch modelling within item response theory.

In the study by Leung and Tsang [24] item responses did, however, in fact not fit the Rasch model as six items (4, 7, 9, 13, 15, and 16) functioned differentially for parents of primary school children compared to parents of children with ADHD recruited from support groups. These findings are unique to this particular study and although the many items functioning differentially could be construed as sign of poor test quality, we cannot on this basis say whether these problems exist in the other language versions of the PSS as well, as none of the other identified studies have conducted item response analysis or analysis of differential item functioning (DIF) of any kind. Leung and Tsang [24] did not report details on the nature of the bias, nor did the article relay whether (or how) PSS scores were adjusted to account for the differential item functioning. As this study to date represents the only attempt to conduct DIF analyses on the PSS, further research is needed on this issue, and particularly for each of the probable two subscales.

In sum, the previous studies of the PSS demonstrate adequate reliability across different languages versions of the PSS and in differing populations. The dimensionality of the PSS, i.e. whether the PSS should be used as a single scale measuring two aspects of parental stress as a single unidimensional measure or as two separate unidimensional subscales, is still uncertain, as it has not been formally tested. In addition, the psychometric properties of the two possible subscales have not been investigated using the Rasch family of measurement models. Furthermore, most of the existing studies using the PSS are based on relatively small samples and only one previous study has examined the use of the PSS among parents of infants. Thus, the aim of this study is to investigate the psychometric properties of the Danish PSS through Rasch analysis with emphasis on the issues of dimensionality and measurement invariance with a sample of mothers of infants 0–12 months old.

## Methods

### Sample

The study sample consists of 1110 mothers with infants aged 0–12 months and is relatively representable for Danish mothers. The sample was collected through two different studies: (1) Baseline data collected in 2013–2014 from a community setting intervention study of 835 first-time mothers residing in the Central Denmark Region who were assessed two months after giving birth. (2) Data collected in 2013 from a community sample of 275 mothers with infants 0–12 months old recruited through either a website frequently visited by parents of small children in Denmark (Sundhedsplejersken.dk) or routine home visits by 160 health visitors from 16 different municipalities. Ethical approval for the study was obtained from the Central Denmark Region Committee on Health Research Ethics (ref.no. 2012–164), the Danish Data Protection Agency (ref.no. 2012-41-1018), and the internal review board at SFI (now VIVE)

Demographic characteristics are presented in Table 1. We found no differences in demographic characteristics between the two samples.

**Table 1:** Demographic characteristics of the study sample (N = 1110).

	Mean	SD	Range
Mother age (years)	30.62	4.40	18–48
Child age (months)	2.70	2.21	0–12
	n	%	
Boys	553	50	
*Mother’s education*			
Public school (grade 9 or 10)	131	12	
Secondary	741	67	
Short tertiary	89	8	
Long tertiary	149	13	

SD: Standard deviation.

### Instrument

The PSS contains 18 item statements, with 10 statements addressing negative and stressful aspects of parenting and eight statements addressing positive aspects of parenting [1], thus taking into account the dichotomous nature of the parenting experience. The item statements are rated for agreement by parents using a 5-point response scale (1 = strongly disagree, 2 = disagree, 3 = undecided, 4 = agree, 5 = strongly agree). The eight positive items are reversed in the coding of the PSS, and a single parental stress sum score is calculated to indicate the degree of parental stress [1].

#### Translation

The translation of the PSS was conducted according to the methods used in translation and cultural adaptation of psychological and educational tests [34]: two independent translations were created by subject matter experts with Danish as the first language. A consensus version was reached, and this was evaluated by an external clinical expert. The final version was back-translated by a native English speaker with subject matter knowledge, and the back-translation was compared to the original with good results. Face validity was investigated among parents with children of different ages (n = 5). Two items (2 and 11) were singled out as somewhat problematic, as one was hard to understand and the other was considered irrelevant. However, as these items are identical to the original items in their content, and the current study is a validity study, we decided to include them to ascertain their possible role in the instrument.

### Analysis

All Rasch analyses were conducted in the DIGRAM software package [35].

#### Rasch measurement models

The Rasch model (RM) for dichotomous items [36] is the simplest item response theory (IRT) model. The RM has five basic requirements for measurement, with the first four providing criterion-related construct validity according to Rosenbaum’s definition [37–39]: 1) *unidimensionality*; items of a scale measure a single underlying latent construct, 2) *Monotonicity*; expected item scores increase with increasing values on the latent variable, 3) *Local independence* (or no local dependence; LD); item responses are conditionally independent of one another given the latent variable, 4) *Absence of differential item functioning* (no DIF); item responses are conditionally independent of relevant background variables (i.e. exogenous variables) given the latent variable, 5) *Homogeneity*; the rank order of item parameters (item “difficulties”) is the same for all persons regardless their level on the latent variable. Fulfillment of these requirements by a set of items means that the sum score is a sufficient statistic for the estimated latent variable. Sufficiency is particularly desirable when one wishes to use the summed raw score of a scale such as it is the case with the PSS, and this property distinguishes scales fitting Rasch models from scales fitting other IRT models [40].

In cases where fit to an RM is rejected, it is still possible to achieve close to optimal measurement, if the departures from the model are only in the form of uniform differential item functioning (uniform DIF) and/or uniform local dependence (uniform LD) [41]. Uniform here simply means that the way items depend either on exogenous variables or other items is the same at all levels of the latent variable. When this is the case, the DIF or LD can be adjusted for in a so-called graphical loglinear Rasch model (GLLRM), as such GLLRMs are simply extensions of the RM allowing precisely the departures of uniform DIF and/or LD. If the GLLRM is only adjusted for uniform LD, the sufficiency of the sum score is not affected, but the reliability of the scale is affected negatively to some degree [41–43]. If the GLLRM is adjusted for uniform DIF, the sum score is no longer a sufficient statistic for the latent variable, as additional information on person’s membership of any subgroups for which items function differentially is needed to obtain the correct sum score. However, when the sum score is equated for DIF this issue is resolved and subsequent comparisons of subgroup scores can be done not confounded by the DIF [44].

#### Item analysis

The distribution of the item responses showed that these were very skewed towards the disagree end of the response scale, as could be expected for the present study sample. Accordingly, the item responses were dichotomized into 0 (strongly disagree and disagree) and 1 (undecided, agree and strongly agree) to achieve a better distribution of the data while keeping the content and meaning of the response categories. Thus, we could proceed with analyses using the dichotomous RM and GLLRM.

It should be noted, that we, in order to be very thorough, did attempt analyses using Masters’ [45] partial credit model (PCM), which is simply a generalization of the RM for ordinal data, and that this was unsuccessful. Thus, the strategy for the dichotomous item analysis was as follows: Initially fit of the full 18-item PSS to the RM was tested and rejected. We then proceeded with the same overall strategy for each of the subscales made up by the negatively and the reversed positively worded items: first, we tested fit of the item responses to the RM, and if this was rejected, we then proceeded to catalogue the departures and subsequently to test the fit of the item responses to a GLLRM adjusting for the departures discovered if these consisted only of uniform LD and/or DIF. When fit to a GLLRM was not achieved, we eliminated the most problematic item (statistically and content-wise problematic) and proceeded again to test fit to the RM and so on.

Overall tests of fit (i.e. comparison of item parameters in low and high scoring groups) and the overall test of no DIF were conducted using Andersen’s [46] conditional likelihood ratio test (CLR). The fit of individual items was tested by comparing the observed item-rest-score correlations with the expected item-rest-score correlations under the model (i.e. the specified RM or GLLRM) [47] as well as conditional infit and outfit statistics [35,48]. The presence of LD and DIF in GLLRMs was tested using conditional tests of independence using Goodman-Kruskal gamma coefficients for the conditional association between item pairs (presence of LD) or between items and exogenous variables (presence of DIF) given the rest-scores [43]. Evidence of overall fit and no DIF was rejected if this was not supported by evidence of individual item fit and lack of evidence of both LD and DIF, in line with the recommendations in [40]. The Benjamini-Hochberg procedure was used to adjust for false discovery rate (FDR) due to multiple testing, whenever appropriate [49]. As recommended by Cox et al. [50], we did not use a critical limit of 5% for p-values as a deterministic decision criterion.

Unidimensionality was assessed through a test of equality of the observed correlation of the two subscales with the expected correlation under the assumption that they measured one and the same latent variable [51], and we used parametric bootstrapping for exact p-values.

In GLLRMs reliability was estimated using Hamon and Mesbah’s [52] Monte Carlo method, as it takes into account any LD in a model and adjusts the reliability accordingly. Accordingly, the reliabilities reported has an interpretation similar to Cronbach’s alpha. Targeting was assessed graphically by plotting the distribution of person parameter locations against the distribution of the item thresholds, as well as by two indices. The graphical representations of targeting allow evaluation as to whether the persons in the study population are included in the range of item parameters on a purely visual basis. The calculated targeting indices allows for numerical evaluation of targeting [48]: the test information target index is the mean test information divided by the maximum test information for theta, and the root mean squared error (RMSE) target index is the minimum standard error of measurement divided by the mean standard error of measurement for theta. Both indices should preferably have a value close to one. Further, we estimated the target of the observed score and the standard error of measurement of the observed score (SEM).

#### Exogenous variables

Parental stress is beside child and parent-child relationship factors related to parental factors such as age, gender, psychopathology, drug abuse, temperament, personality and social conditions [15]. To examine measurement invariance (i.e. absence of DIF we included two exogenous parental variables of importance for the assessment of maternal stress. Specifically, we tested for the presence of DIF relative to the age and educational level of the mothers.

Being a very young mother (<20 years) is related to adverse health, economic, and social outcomes [53,54]. In Denmark, there are few teenage mothers (1% of all births in 2013), so mothers in the age 20–24 are also considered young (11% of all births in 2013). According to Statistics Denmark, the mean age for Danish mothers is 30.9 (29.1 for first-time mothers). Being a relatively old mother can also be problematic as there is evidence that having a child in advanced (35–37 years old) or very advanced age (≥38) for some mothers can be related to low level of education, unemployment, single status, unplanned pregnancy and unsatisfactory relationship with the partner [55]. In accordance with this, the association between parental stress and age seem to be curvilinear, with very young mothers and older mothers reporting higher parental stress levels than mothers in their late twenties and early thirties [56]. As there may be challenges related to both being a relatively young and a relatively old mother, but we could not define further the true cut points dividing Danish mothers into “young, normal and old”, we instead split the sample at the “mean birth age” for Danish women, creating two groups of mothers for DIF analysis; a group of younger mothers (<30) and a group of older mothers (≥30).

Growing up in a family with persistent socioeconomic disadvantage is a serious risk factor for children [57]. Single parents and households where parents have low or no education are at greater risk for experiencing socioeconomic disadvantages. Low education has been associated with higher parental stress, but as with age the relationship is not strictly linear but, as in the case of age, seem to be curvilinear with both mothers with low education and mothers with high education experiencing higher levels of parental stress than mothers with intermediate education [58,59]. To examine the relationship between parental stress and level of education we split the sample into two groups for DIF analysis, thus creating a group of mothers with short education (secondary schooling or less) and mothers with long education (tertiary education).

The distribution of the mothers in age and educational groups for DIF analysis are shown in Table 2.

**Table 2:** Distribution of mothers in age and education level groups for differential item function analysis.

	N	%
age < 30	464	42
age ≥ 30	646	58
N/%	1110	100
Edu ≤ secondary	872	89
Edu ≥ short tertiary	238	21
N/%	1110	100

## Results

Analysis of the original full 18-item PSS showed that it did not fit the RM (overall fit statistics are provided in S1 Table in the supplement), nor was it possible to obtain fit to a GLLRM with all 18 items. Further analysis showed that in order to establish fit to any model, several items had to be eliminated. Incidentally, the set of remaining items and the set of eliminated items were more or less the same sets of items which had been proposed to be subscales in previous research [1,23,25]. Accordingly, we proceeded with separate analyses of items belonging to the two subscales Parental Stress and Lack of Parental Satisfaction (Table 3).

**Table 3:** The PSS items divided into the two proposed subscales; parental stress and lack of parental satisfaction.

Item	**Parental Stress subscale (PS)**
3	Caring for my child(ren) sometimes takes more time and energy than I have to give
4	I sometimes worry whether I am doing enough for my child(ren)
9	The major source of stress in my life is my child(ren)
10	Having child(ren) leaves little time and flexibility in my life
11	Having child(ren) has been a financial burden*
12	It is difficult to balance different responsibilities because of my child(ren)
13	The behavior of my child(ren) is often embarrassing or stressful to me
14	If I had it to do over again, I might decide not to have child(ren)
15	I feel overwhelmed by the responsibility of being a parent
16	Having child(ren) has meant having too few choices and too little control over my life
	**Lack of Parental Satisfaction (LPS)–reversely scored items**
1	I am happy in my role as a parent
2	There is little or nothing I wouldn't do for my child(ren) if it was necessary*
5	I feel close to my child(ren)
6	I enjoy spending time with my child(ren)
7	My child(ren) is an important source of affection for me
8	Having child(ren) gives me a more certain and optimistic view for the future
17	I am satisfied as a parent
18	I find my child(ren) enjoyable

*: items excluded from final models

Neither of the two proposed subscales; the 10-item Parental Stress subscale, and the 8-item Lack of Parental Satisfaction scale fit Rasch models, nor was it possible to establish fit to a GLLRM for any of the two subscales. Upon elimination of the worst fitting item in each subscale (i.e. PS item 11 *Having child(ren) has been a financial burden and* LPS item 2 *There is little or nothing I wouldn't do for my child(ren) if it was necessary* and), still none of the scales fit Rasch models, as evidence of departures in the form of DIF and local dependence between items were found. Subsequently, each of the subscales fitted GLLRMs, though of differing complexity. Global tests-of-fit and differential item function for the two subscales are presented in Table 4.

**Table 4:** Global tests-of-fit and differential item function for the parental stress and the lack of parental satisfaction subscales to Rasch models and the graphical loglinear Rasch models in Figs 1 and 2.

Tests	PS (RM)			PS (GLLRM)^a^			LPS (RM)			LPS (GLLRM)^b^		
	*CLR*	*df*	*P*	*CLR*	*Df*	*P*	*CLR*	*df*	*p*	*CLR*	*df*	*P*
Global homogeneity	27.9	8	< .01	17.6	15	.28	20.0	6	< .01	14	8	.08
*DIF relative to*:												
Mothers’ age	52.7	8	< .01	16.8	13	.21	14.9	6	< .05	20.1	8	.01
Mothers’ education	22.0	8	< .001	21.0	11	.03	14.6	6	< .05	16.2	8	.04

PS: Parental stress; RM: Rasch model; GLLRM: Graphical loglinear Rasch model; LPS: Lack of parental satisfaction; CLR: Conditional likelihood ratio; df: degrees of freedom; p: p-value; DIF: differential item function.

Global homogeneity test compares items parameters in approximately equal-sized groups mothers scoring low and high. The critical limits for the p-values related to the two GLLRMs (a and b) after adjusting for FDR were: 5% limit p = .0167, 1% limit p = .0033, thus providing no solid evidence of further DIF.

^a^ The model for the PS scale assumes that some items pairs are locally dependent (items 3 and 4, 9 and 10, 10 and 16, and 12 and 16), that item 16 functions differentially relative to the mothers’ educational level and age, and that item 3 also functions differentially relative to the mothers’ age.

^b^ The model for the LPS subscale assumes that items 1 and 17, and 17 and 18 are locally dependent.

**Fig 1:**
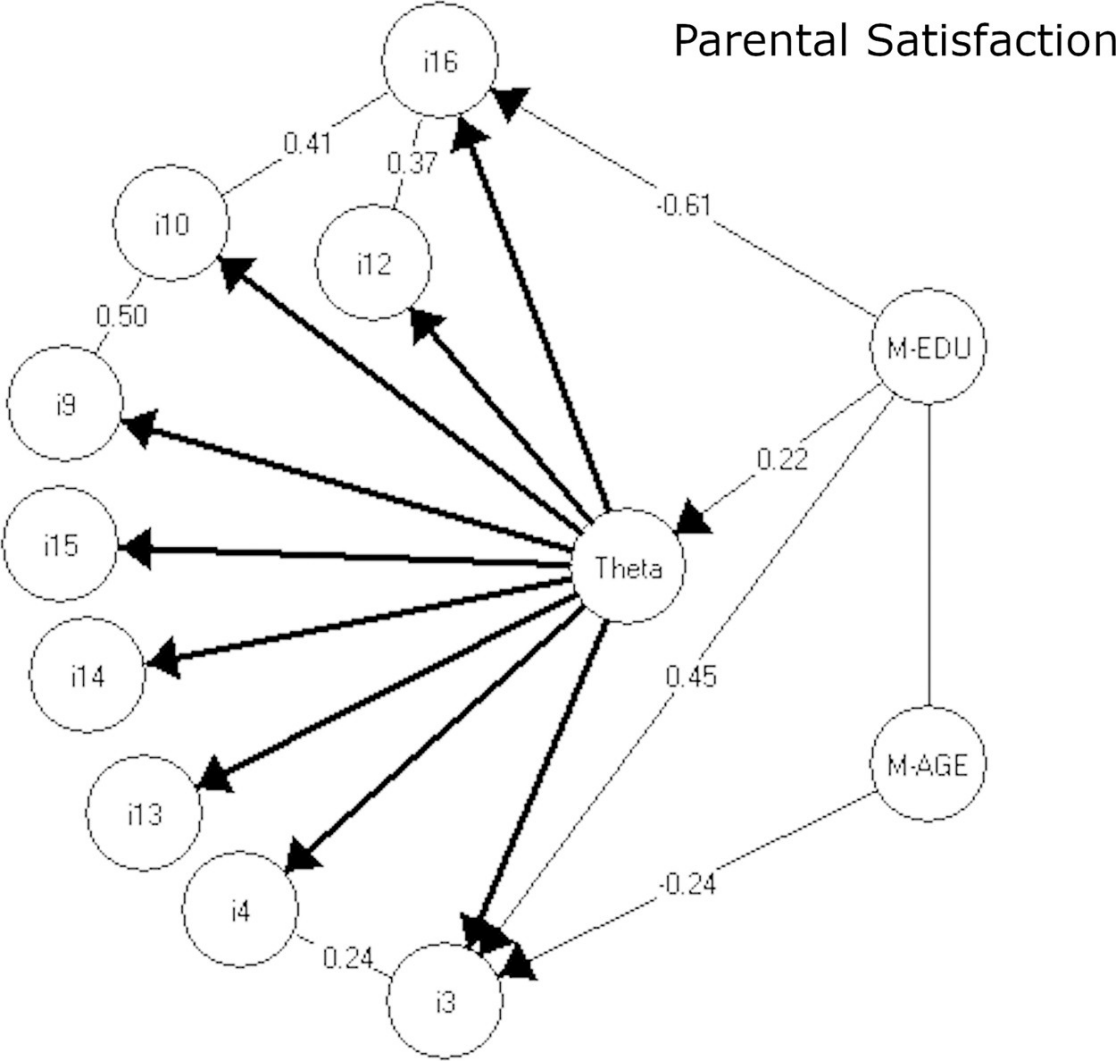
The resulting graphical loglinear Rasch models for the parental stress subscale. Note: Theta is the latent parental stress score.

**Fig 2:**
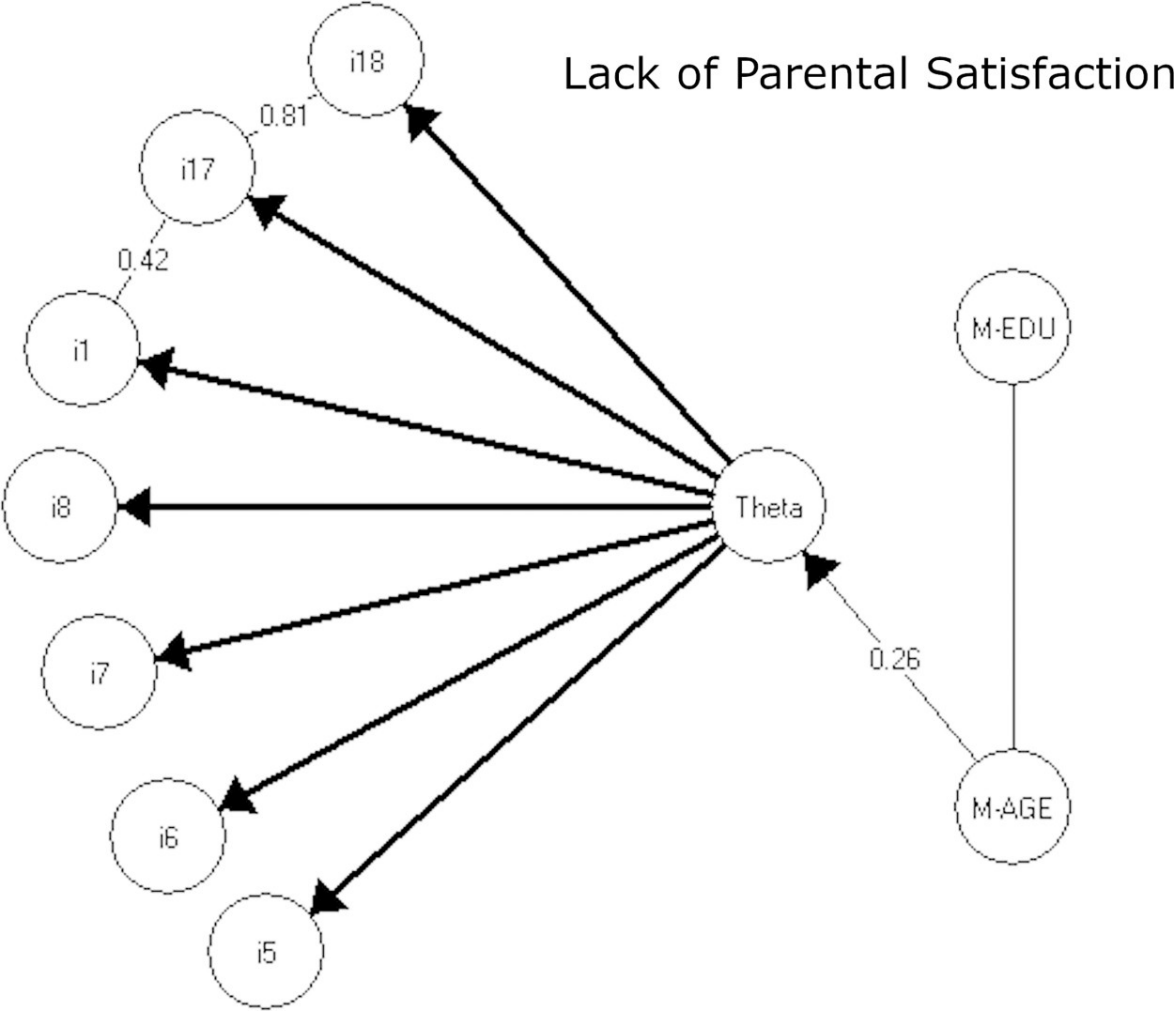
The resulting graphical loglinear Rasch models for the lack of parental satisfaction subscale. Note: Theta is the latent lack of parental satisfaction score.

The 9-item PS subscale fits a GLRRM with both local dependences between six out of the nine items, as well as DIF for two items. The six locally dependent items were: items 3 (*Caring for my child(ren) sometimes takes more time and energy than I have to give*) and 4 (*I sometimes worry whether I am doing enough for my child(ren)*), item 9 (*The major source of stress in my life is my child(ren)*) and 10 (*Having child(ren) leaves little time and flexibility in my life*), item 10 and 16 (*Having child(ren) has meant having too few choices and too little control over my life*), and item 16 and 12 (*It is difficult to balance different responsibilities because of my child(ren)*). Two PS-items were also found to function differentially: item 16 suffered from DIF relative to maternal education, so that mothers with short education were systematically more likely to agree that *having child(ren) has meant having too few choices and too little control over my life*, than were mothers with longer education, no matter their level of parental stress. Item 3 suffered from DIF relative to both maternal education and maternal age. Accordingly, mothers with short education were systematically *less* likely to agree that *caring for my child(ren) sometimes takes more time and energy than I have to give*, than were mothers with longer education, while mothers below 30 years of age were *more* likely to agree with the item statement compared to mothers aged 30 years and older, no matter their level of parental stress.

The 7-item LPS subscale fit a GLRRM with strong local dependence between items 1 (*I am happy in my role as a parent*) and 17 (*I am satisfied as a parent*), and item 17 and 18 (*I find my child(ren) enjoyable*). No evidence of DIF was found for any of the LPS items.

### The effect of DIF in the parental stress subscale

Two items in the PS subscales suffered from DIF relatively to maternal age and education. Thus, to be able to use the summed PS scale score in the subsequent statistical analysis, the PS score had to be equated for this DIF, in order to eliminate any confounding effect of the DIF. A score equation table is provided in the supplement for this purpose (S2 Table). The question is to which degree the DIF would confound statistical analysis or lead to erroneous clinical decisions if not dealt with adequately. If conducting a simple comparison of mean PS scores for mothers with secondary education or less compared to mothers with short tertiary education or longer, it does not make much of a difference whether the comparison is made with summed raw scores (i.e. observed scores) or the scores adjusted for DIF (Table 5). However, it must be taken into account that this is partially due to the fact that both groups are adjusted due to the additional age DIF. The opposite is the case if conducting a similarly simple comparison of mean PS scores for mothers below 30 years of age compared to mothers aged 30 or more. In this case, the conclusion reached on the basis of the scores adjusted for DIF (Table 5) would differ substantially from the conclusion reached on the basis of comparing the observed scores. In fact, if p-values are evaluated using Cox et al.’s [50] recommendations, rather than employing a deterministic 5% level of significance, a comparison of the observed PS scores would lead to a type II error (i.e. incorrectly retaining the false null-hypothesis of equality). Accordingly, score equation to adjust for DIF should be undertaken, if employing the summed scale score of the PS subscale for research purposes. However, for clinical purposes (i.e. assessing individual mothers), it appears that adjusting for DIF would not change any classification if a standard rule of rounding scores is applied (S2 Table). As such the equating of scores does not have any immediate implication for the clinical and practical use of the PS subscale.

**Table 5:** Comparison of observed and equated mean parental stress scores in education and age groups.

	N	Observed scores		Adjusted scores		Bias
		*Mean*	*SE*	*Mean*	*SE*	
*Education*^*1*^						
Edu ≤ secondary	872	3.10	0.07	3.16	0.07	.06
Edu ≥ short tertiary	238	3.84	0.13	3.91	0.14	.07
*Age*^*2*^						
Age<30	464	3.11	0.10	3.11	0.10	.00
Age≥30	646	3.37	0.08	3.47	0.08	.10

SE: Standard error.

^1^ Differences in observed mean scores (χ^2^ (1) = 24.1, p < 0.001). Differences in adjusted mean scores (χ^2^ (1) = 23.1, p < 0.001)

^2^ Differences in observed mean scores (χ^2^ (1) = 4.6, p = 0.032). Differences in adjusted mean scores (χ^2^ (1) = 8.3, p = 0.004)

### Targeting and reliability

**Fig 3:**
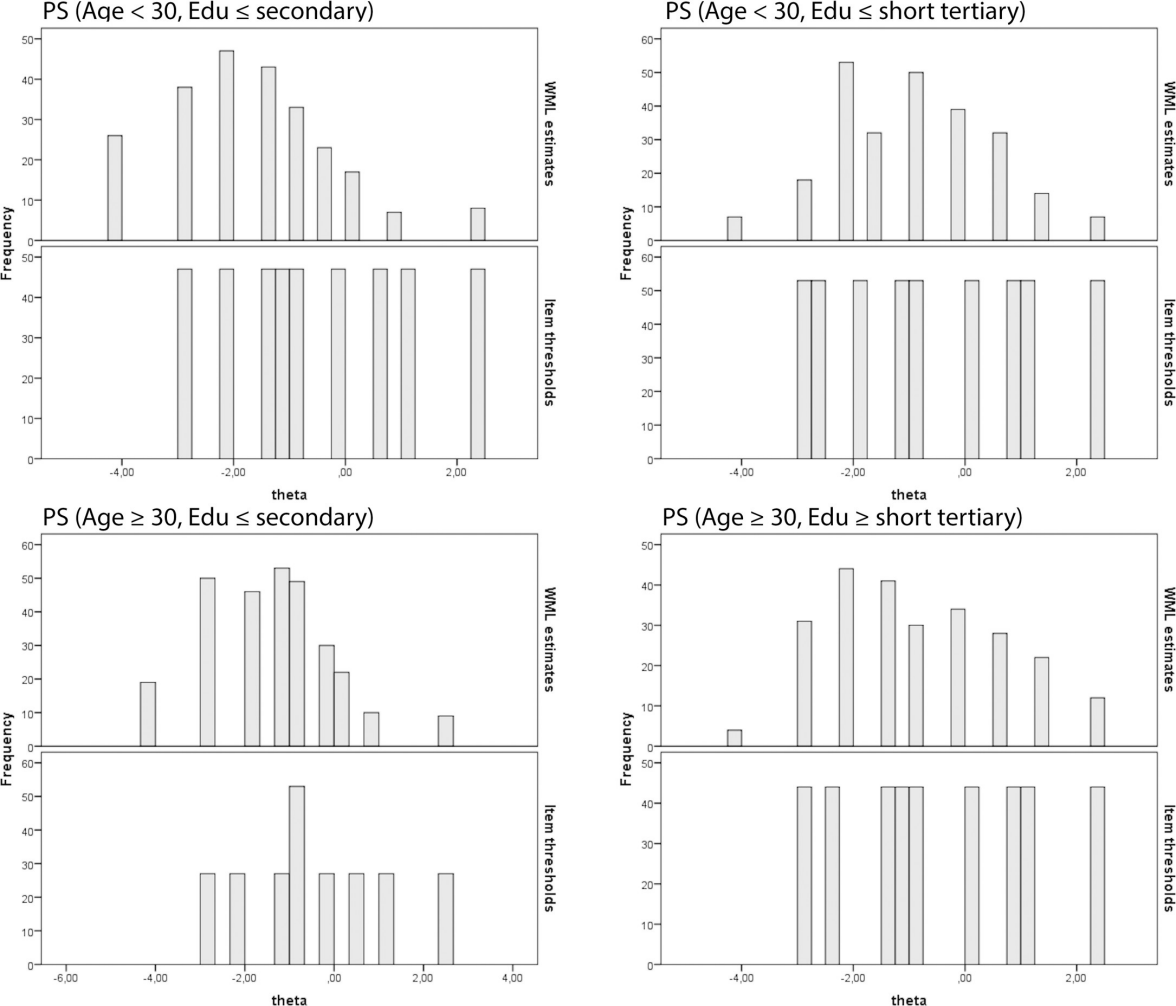
Targeting of person parameter locations and items thresholds along the latent PS scale for groups of mothers defined by age and educational level (DIF-variables). Note: WML estimates: person estimates on the latent parental stress scale (theta). Item thresholds: item difficulties on the latent parental stress scale (theta).

Targeting of the PS and LPS subscales differed substantially (Table 6). Targeting of the PS scale was excellent, though some variation was found across the four groups of mothers defined by their age and educational level. Thus, the best targeting of the PS was found for mothers below 30 years of age with a long education (with 91% of the maximum obtainable information achieved) and the least good for mothers with a short education irrespective of their age (78% and 80% of the maximum obtainable information, respectively). The less good targeting for mothers with short education is caused by the person parameters for these mothers being located slightly more towards the lower end of the latent PS scale, while the item parameters were located slightly more toward the high end of the PS scale (e.g. some mothers were showing lower levels of parental stress than the levels needed to endorse even the easiest items) (Fig 3).

**Table 6:** Targeting and reliability of the parental stress and lack of parental satisfaction subscales.

	Theta								Sum score			
Groups of mothers defined by DIF^a^	Target	Mean	TI mean	TI max	TI Target index	RMSE mean	RMSE min	RMSE target index	Target	Mean	Mean SEM	r
**Parental Stress (PS)**												
Age below 30 & short education (n = 393)	-.80	-1.63	1.338	1.719	.778	.844	.763	.903	4.15	2.98	1.14	.67
Age below 30 & long education (n = 71)	-.80	-1.03	1.340	1.472	.910	.859	.824	.959	4.11	3.82	1.15	.64
Age 30 and above & short education (n = 479)	-.71	-1.34	1.413	1.764	.801	.838	.753	.899	4.18	3.21	1.18	.65
Age 30 and above & long education (n = 167)	-.84	-.96	1.315	1.538	.855	.865	.806	.932	3.96	3.85	1.14	.71
**Lack of Parental Satisfaction (LPS)**												
All *(no DIF)*	-.40	-4.72	.312	1.537	.203	1.625	.807	.496	3.48	.54	.49	.61

TI: Test information; RMSE: The root mean squared error of the estimated theta score; SEM: The standard error of measurement of the observed score; r: Reliability.

^a^. For the PS subscale targeting and reliability is provided for groups defined by DIF variables.

**Fig 4:**
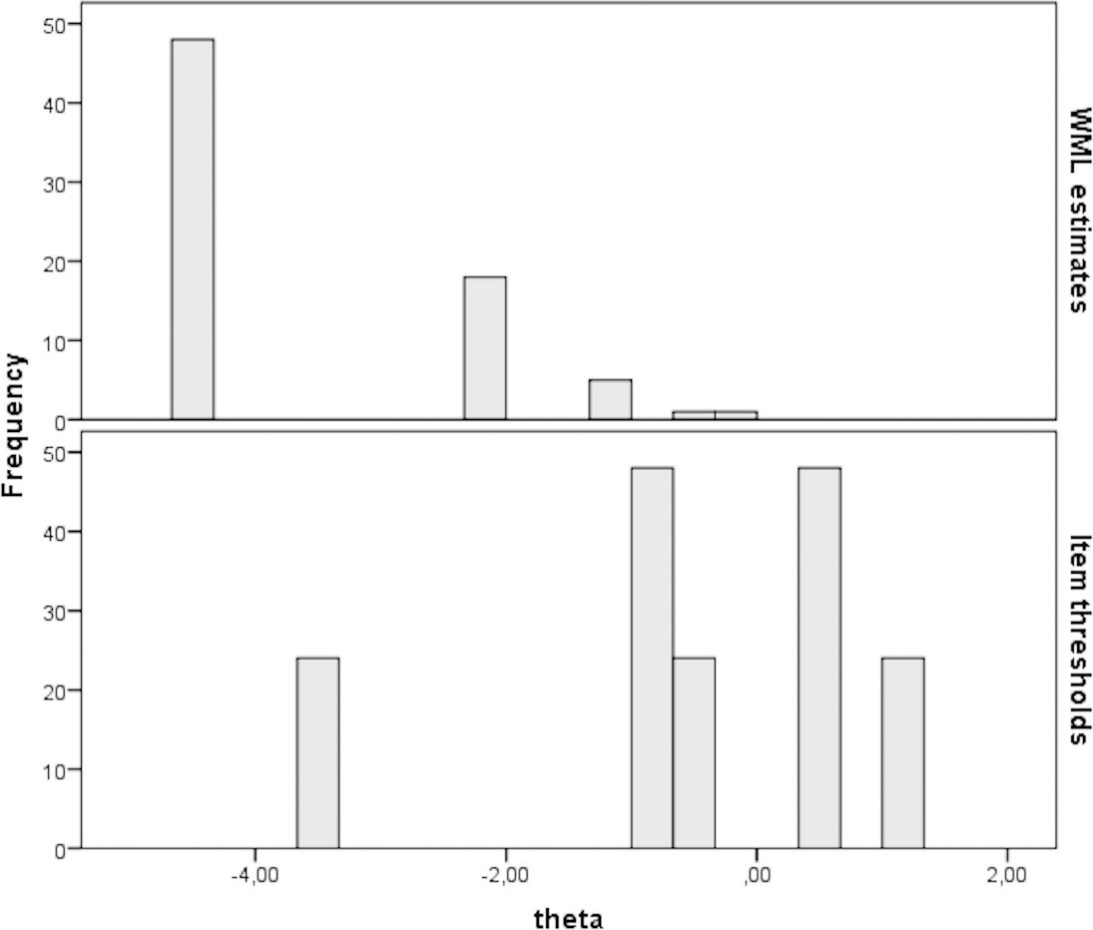
Targeting of person parameter locations and items thresholds along the latent LPS scale for all mothers. Note: WML estimates: person estimates on the latent lack of parental satisfaction scale (theta). Item thresholds: item difficulties on the lack of parental satisfaction scale (theta).

The targeting of the LPS scale was very poor (20% of the maximum obtainable information was achieved). This very poor targeting was caused by the fact that the person parameters for the majority of the mothers were located at the low end of the LPS scale (e.g. they did not lack parental satisfaction), while the item parameters (e.g. the item difficulties denoting how lacking a mother should be in parental satisfaction in order to endorse the different items) were located more toward the high end of the LPS scale (Fig 4).

The reliability of both the PS and the LPS were less than optimal, with the highest reliability for the group of mothers aged 30 years or older with a long education (Table 6).

### Unidimensionality

Following the establishment of fit to GLLRMS for both the PS and the LPS subscales, we proceeded to test the null-hypothesis that these in fact measured one and the same latent construct (i.e. parental stress), as opposed to the two separate latent constructs (i.e. parental stress and lack of parental satisfaction). Both the asymptotic and the parametric bootstrapping test clearly rejected unidimensionality (observed gamma between subscale scores .295, expected gamma between subscale scores .494, s.e. = .03, asymptotic p < .001, exact p < .0001). We, therefore, maintain the original notion of Berry and Jones [1] that the PSS, in fact, measure parental stress and parental satisfaction (or parental satisfaction if not reversed, if preferred by clinicians), but further suggest that this should be done separately by two unidimensional scales. Accordingly, we also suggest that reducing the two scales to a single total scale and one total score would be invalid. For completeness, we tested the overall fit to the common 16-item GLLRM resulting from combining the PS and LPS subscale GLLRMs, and this was clearly rejected (S3 Table in the supplement).

### Parental stress versus lack of parental satisfaction

**Fig 5:**
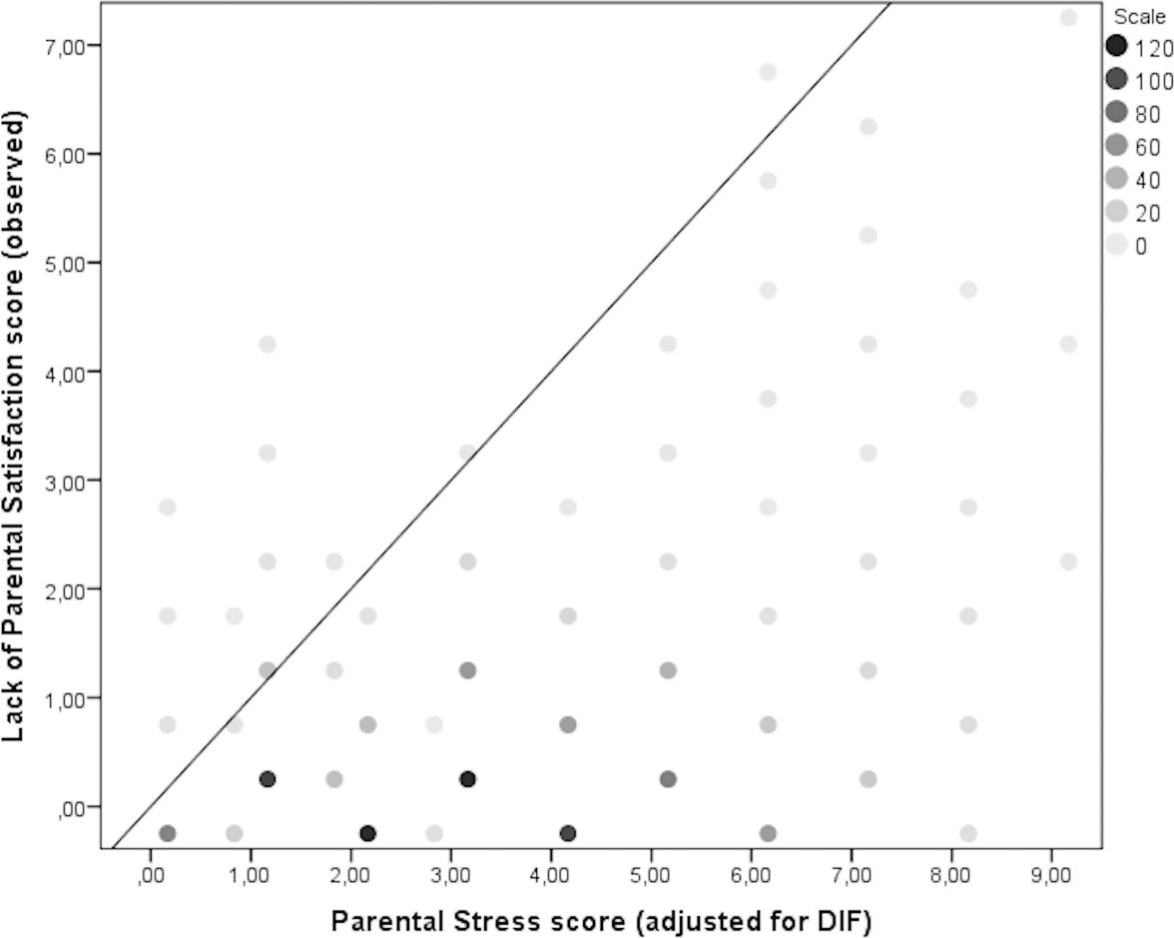
Distribution of parental stress and lack of parental satisfaction sum scores.

Having established that the PSS is made up of two unidimensional and not very strongly correlated subscales (c.f. the above sections) measuring parental stress (PS) and lack of parental satisfaction (LPS), a plot of the PS and LPS score distributions shown in Fig 5 illustrates the value of considering these as separate constructs. Most mothers were located in the lower left quadrant of the plot and thus were scoring low on both lack of parental satisfaction and parental stress. However, it was also evident that a substantial number of mothers scored relatively high on parental stress while scoring low on lack of parental satisfaction (i.e. were satisfied as parents).

## Discussion and implications

In this study, we examined the validity of the 18-item Parental Stress Scale using Rasch models and graphical loglinear Rasch models. The results of the analyses showed that the PSS is not a single unidimensional scale, but rather consists of two separate unidimensional subscales measuring parental stress (PS) and lack of parental satisfaction (LPS). The PS and the LPS subscales both fit graphical loglinear Rasch models (GLLRM), with departures from theRasch model in the form of local dependence between some items (LD), and in the case of the PS subscale also in the form of differential item functioning (DIF). However, all LD and DIF departures were uniform in nature, and could easily be adjusted for to make both the latent scales scores (i.e. person parameters) and the observed scale scores (i.e. the summed raw scores) comparable across subgroups. We found further support for the two separate constructs when plotting the subscales scores against one another. The targeting of the PS subscale to the population in the study was excellent, while the targeting of the LPS subscale was very poor. Taken together, the findings supported both the construct validity of the parental stress construct as a dichotomy of stressful and satisfying experiences as originally suggested by Berry and Jones. The current results though, also show that this should actually be measured as a dichotomy (i.e. by scoring and interpreting the PSS as two separate subscales).

Reliability of the two subscales was acceptable for use in large-scale research studies, but in the low end for use as a screening tool at the individual level with this particular sample (parental stress 0.64–0.71; lack of parental satisfaction 0.61). Reliability tends to decrease with fewer items and with the number of response categories for each item, and we, therefore, expected a lower reliability for the subscales compared to the reliability for the total scale as is reported in most of the previous studies. This is also what we found. Two previous studies have reported reliability for two subscales, though the items that the two subscales consist of across these studies and the current study are not exactly identical. In the Spanish study by Oronoz and colleagues reliabilities were reported as 0.76 for seven (3,9,10,11,12,13,15) of the nine items found to make up the PS subscale in the current study) and 0.77 for five (1,5,6,16,17) of the seven items found to make up the LPS subscale in the current study [25]. In the Portuguese study by Brito and Faro reliabilities were reported as 0.79 for eight items (3,9,10,11,12,14,15,16), seven of which overlaps items in the PS subscale in the current study, and 0.69 for eight items (1,5,6,7,8,13,17,18), seven of which correspond to the LPS subscale in the current study [26]. It is also highly plausible that some of the reduction in reliabilities in the current study compared to the other two studies is due to the local dependence between items in both subscales, as this is known to produce inflated Cronbach alpha values when not adjusted for in the calculation. We thus recommend that the reliabilities are evaluated for other populations prior to using the scale for screening purposes.

The targeting, or the extent to which items in the two subscales match the study population, was excellent for the PS subscale, but less than optimal for the LPS subscale. The poor targeting of the LPS subscale means that the measurement of lack of parental satisfaction in this particular group of mothers of infant has less precision than we could have wished for, as the mothers are located in a part of the scale where there is least information. However, the finding was not surprising as most mothers of infants find it satisfying and rewarding to be a mother despite the hardship that is also an inevitable part of being a parent. Thus, we recommend that future validity studies of the two subscales in the PSS include a wider range of parents, for example parents with risk factors such as mental health problems, drug abuse, poverty, or parents of children with for example behavior problems, depression or anxiety, in order to get a broader assessment of the targeting of the LPS subscale in particular, as this might differ (i.e. be better) in a different population.

The elimination of item 2 *(There is little or nothing I wouldn't do for my child(ren) if it was necessary)* from the LPS subscale in the current study corresponds well with findings in all the identified previous PSS validity studies, which all eliminated this item [1,23,25,26]. As discovered already in our face validity investigation, this item is very hard to understand, and thus it was no surprise that the Rasch analysis showed that this item did not fit any of the scales. The elimination of item 11 *(Having child(ren) has been a financial burden)* in the current study, is only supported by the original validity study by Berry and Jones [1], as the other four identified studies do not eliminate item 11. As the identified validity studies [1,23,25,26] have been conducted in very different cultural settings, we can only speculate whether this discrepancy might stem from cross-cultural differences in family affluence, from culturally determined perceptions of whether children can be seen as a financial burden or other related differences. Future cross-cultural studies using the PSS might successfully be designed to explore this issue.

The majority of the mothers seem to be well functioning and have low levels of parental stress and high levels of parental satisfaction. Only a few mothers report high levels of parental stress and low parental satisfaction. This is probably because it is a relatively representative sample and we would not expect to find many mothers struggling with parental stress and low parental satisfaction. Very few mothers express lack of parental satisfaction. This is consistent with the sample consisting of mainly first-time mothers of infants that are generally express parental satisfaction i.e. that they enjoy being a mother, feel connected to and loved by the infant.

It is common for mothers of infants to feel a bit stressed due to the demands of taking care of an infant such as lack of sleep and handling a fussy or crying infant. In line with this, we do find a substantial number of mothers who report high levels of parental stress combined with high levels of parental satisfaction. It is positive that these mothers experience a high level of parental satisfaction. This may, however, decrease over time if the parental stress level remains high. During the first year of the infant’s life, the majority of Danish mothers are on maternity leave. Some mothers seem to find it very satisfactory but also stressful to be a stay-at-home parent. Most children are the results of planned pregnancies. New parents have high expectations about becoming a parent and have access to unlimited information on how to take care of the infant. This may cause mothers who are inclined to worry about being a good enough parent to worry even more and feel inadequate as a mother.

Based on the results, we recommend that with populations similar to the one in the present study the Danish version of the PSS should be administered in the 16 item version with the original response categories, but scored according to the dichotomized response categories, as this will allow respondents to provide differentiated answers as well as valid scoring. It is possible that the original response categories can be used with a more diverse sample. We furthermore recommend that the PSS is scored and interpreted as two separate subscales (PS and LPS), as a single total score is not a valid measure in itself for this population. For use in clinical settings, we consider it an advantage that the PSS consists of two subscales measuring different aspects of parenting as this provides more information on how the parent is feeling. It is likely that a high level of parental satisfaction can act as a buffer so that it is more concerning if a parent scores high on parental stress and low on parental satisfaction compared to a parent scoring high on both parental stress and parental satisfaction. However, future studies should address the issue of setting appropriate clinical or intervention cut points for both scales, preferably in the form of a sensitivity-specificity study.

A strength of this study is that we conducted Rasch analyses with a large and highly compatible community sample of mothers of infants. This is ideal for validity purposes, as we thereby control the number of variations. Further strengths are the formal testing of differential item functioning, local dependence among items, and unidimensionality of the two subscales while taking into account any LD and DIF in these. A limitation of the study is that the sample is relatively representative, but does not include mothers of children with identified problems or diagnoses. Hence future validity studies should include mothers/parents to children from such groups. Furthermore, the sample only includes mothers, as the intervention study from which the majority of the data was drawn was aimed at only mothers and as we received very few responses from fathers in the web-survey. Thus, as previous studies also mostly included mothers, further studies on the psychometric properties of the PSS when used with fathers and parent couples are still needed. The sample predominantly consists of first-time mothers. As it is likely that mothers with more than one child experience different levels of parental stress than do first-time mothers, this should be examined in future validity studies of the PSS. Future studies should also include parents of older children as parental demands vary across the course of childhood.

## Conclusion

In conclusion, we found that the PSS is not a single unidimensional scale but rather consists of two separate unidimensional, construct-valid subscales measuring parental stress (PS) and lack of parental satisfaction (LPS), in accordance with the original theoretical proposal by Berry and Jones [1]. The PS and the LPS subscales, can when appropriately adjusted for the bias caused by differential items functioning, be used to assess the level of parental stress and any lack of parental satisfaction experienced by mothers of infants in the general population, with an acceptable level of precision.

## Availability of the Danish PSS

The Danish PSS is shown in appendix 1 and is available to researchers and clinicians free of charge upon request. Please contact the corresponding author.

## Appendix 1

### Oplevelse af forældreskab

Nedenfor er en række udsagn, som beskriver forskellige følelser og oplevelser i forbindelse med det at være forælder.

Vurder hvert enkelt udsagn ud fra hvordan dit forhold til dit barn/dine børn generelt er.

Angiv med et tal på linjen ud for hvert enkelt udsagn hvor enig eller uenig du er i udsagnet vha. nedenstående svar-skala:

**1 = meget uenig 2 = uenig 3 = hverken uenig eller enig 4 = enig 5 = meget enig**

____ 1. Jeg nyder min rolle som forælder.

____ 2. Der er meget lidt jeg ikke vil gøre for mit barn/mine børn, hvis det er nødvendigt

____ 3. Det kræver nogle gange mere tid og energi end jeg har at tage mig af mit barn/mine børn.

____ 4. Jeg bekymrer mig nogle gange over om jeg gør nok for mit barn/mine børn.

____ 5. Jeg føler mig tæt forbundet med mit barn/mine børn.

____ 6. Jeg nyder at tilbringe tid sammen med mit barn/mine børn.

____ 7. Mit barn/mine børn er en vigtig kilde til kærlighed for mig

____ 8. At have et barn/børn giver mig et mere trygt og optimistisk syn på fremtiden.

____ 9. Den største kilde til stress i mit liv er mit barn/mine børn.

____ 10. At have et barn/børn giver mig begrænset tid og fleksibilitet i mit liv

____ 11. At få et barn/børn har været en for stor pengemæssig byrde.

____ 12. At have et barn/børn, gør det svært at få mine forskellige ansvar og forpligtelser til at gå op.

____ 13. Mit barn/mine børn opfører sig ofte på måder som gør mig flov eller stresser mig.

____ 14. Hvis jeg skulle gøre det om, ville jeg måske vælge ikke at få børn.

____ 15. Jeg føler mig overvældet af det ansvar det er at være forælder.

____ 16. At have et barn/børn har betydet at jeg har haft for få valgmuligheder og for lidt kontrol over mit liv.

____ 17. At være forælder tilfredsstiller mig.

____ 18. Jeg har stor glæde af mit barn/mine børn.

## Supporting information

S1 Table Global Tests-of-fit for the original 18-item parental stress scale to the Rasch model. (DOCX)

S2 Table Score equation to adjust for education and age differential item function in the parental stress subscale. (DOCX)

S3 Table Global Tests-of-fit for the 16-items from the resulting parental stress and lack of parental satisfaction subscales to the common graphical loglinear Rasch model. (DOCX)
