# Supplementary material for: Psychometric properties of the Danish Parental Stress Scale: Rasch analysis in a sample of mothers with infants
Source: PLoS One. 2018 Nov 7;13(11):e0205662. doi: 10.1371/journal.pone.0205662 (PMC6221275; doi:10.1371/journal.pone.0205662)
Supplement: S1 Table — (DOCX) [file pone.0205662.s001.docx]

S1 Table. Global Tests-of-fit for the original 18-item parental stress scale to the Rasch model

| Tests | 18-item PSS (RM) | | |
| --- | --- | --- | --- |
|  | *CLR* | *Df* | *p* |
| Global homogeneity | 88.7 | 17 | < .001 |
| *DIF relative to* |  |  |  |
| Mothers’ age | 88.8 | 17 | < .001 |
| Mothers’ education | 61.4 | 17 | < .001 |

RM: Rasch model; CLR: Conditional likelihood ratio.

Global homogeneity test compares items parameters in approximately equal-sized groups mothers scoring low and high. The critical limit for the p-values after adjusting for FDR remained to be .05 at the 5% level, thus not affecting significance.
