# Supplementary material for: Psychometric properties of the Danish Parental Stress Scale: Rasch analysis in a sample of mothers with infants
Source: PLoS One. 2018 Nov 7;13(11):e0205662. doi: 10.1371/journal.pone.0205662 (PMC6221275; doi:10.1371/journal.pone.0205662)
Supplement: S2 Table — (DOCX) [file pone.0205662.s002.docx]

S2 Table. Score equation to adjust for education and age differential item function in the parental stress subscale

| Observed score^1^ | Equated scores | | |
| --- | --- | --- | --- |
| Age < 30 | Age < 30 | Age ≥ 30 | Age ≥ 30 |
| Edu ≤ secondary | Edu ≥ short tertiary | Edu ≤ secondary | Edu ≥ short tertiary |
| 0.00 | 0.00 | 0.00 | 0.00 |
| 1.00 | 0.88 | 1.06 | 0.97 |
| 2.00 | 1.82 | 2.12 | 1.97 |
| 3.00 | 2.87 | 3.15 | 3.02 |
| 4.00 | 4.01 | 4.14 | 4.13 |
| 5.00 | 5.17 | 5.11 | 5.23 |
| 6.00 | 6.23 | 6.07 | 6.26 |
| 7.00 | 7.17 | 7.03 | 7.18 |
| 8.00 | 8.06 | 9.01 | 8.06 |
| 9.00 | 9.00 | 9.00 | 9.00 |

^1^ Reference group. Edu: Education group
