# Supplementary material for: Psychometric properties of the Danish Parental Stress Scale: Rasch analysis in a sample of mothers with infants
Source: PLoS One. 2018 Nov 7;13(11):e0205662. doi: 10.1371/journal.pone.0205662 (PMC6221275; doi:10.1371/journal.pone.0205662)
Supplement: S3 Table — (DOCX) [file pone.0205662.s003.docx]

S3 Table. Global Tests-of-fit for the 16-items from the resulting parental stress and lack of parental satisfaction subscales to the common graphical loglinear Rasch model

| Tests | 16-item PS+LPS (GLLRM)^a^ | | |
| --- | --- | --- | --- |
|  | *CLR* | *Df* | *P* |
| Global homogeneity | 47.0 | 24 | < .01 |
| *DIF relative to* |  |  |  |
| Mothers’ age | 44.8 | 22 | < .001 |
| Mothers’ education | 60.1 | 20 | < .01 |

GLLRM: Graphical loglinear Rasch model; CLR: Conditional likelihood ratio. Global homogeneity test compares items parameters in approximately equal-sized groups mothers scoring low and high. The critical limits for the p-values related to the GLLRM after adjusting for FDR remained to be .05 at the 5% level, thus not affecting significance.

^a^ The model for the common 16-item PS+LPS scale assumes that some items pairs are locally dependent (items 1 and 17, 3 and 4, 9 and 10, 10 and 16, 12 and 16, and 17 and 18), that item 16 functions differentially relative to the mothers’ educational level and age, and that item 3 also functions differentially relative to the mothers’ age.
